# Production and purification of chimeric HBc virus-like particles carrying influenza virus LAH domain as vaccine candidates

**DOI:** 10.1186/s12896-017-0396-8

**Published:** 2017-11-10

**Authors:** Andris Kazaks, I-Na Lu, Sophie Farinelle, Alex Ramirez, Vincenzo Crescente, Benjamin Blaha, Olotu Ogonah, Tarit Mukhopadhyay, Mapi Perez de Obanos, Alejandro Krimer, Inara Akopjana, Janis Bogans, Velta Ose, Anna Kirsteina, Tatjana Kazaka, Nicola J. Stonehouse, David J. Rowlands, Claude P. Muller, Kaspars Tars, William M. Rosenberg

**Affiliations:** 1 Latvian Biomedical Research and Study Centre, Ratsupites 1, Riga, LV-1067 Latvia; 2 Department of Infection and Immunity, Luxembourg Institute of Health, 29, rue Henri Koch, L-4354 Esch-sur-Alzette, Luxembourg; 3 iQur Limited, 2 Royal College Street, London, NW1 0NH UK; 4 Department of Biochemical Engineering, University College London, Gower Street, London, WC1E 6BT UK; 5 3P Biopharmaceuticals SL, Calle Mocholi 2 Poligono Mocholi, Noain, 31110 Navarra, Spain; 6 Faculty of Biological Sciences, University of Leeds, Leeds, LS2 9JT UK; 7 Institute for Liver and Digestive Health, Division of Medicine, University College London, Royal Free Campus, NW3 2PF, London, UK; 8 Faculty of Biology, Department of Molecular Biology, Jelgavas 1, Riga, LV-1004 Latvia

**Keywords:** Virus-like particles, Tandem-core, Influenza vaccine, Long alpha helix, Yeast

## Abstract

**Background:**

The lack of a universal influenza vaccine is a global health problem. Interest is now focused on structurally conserved protein domains capable of eliciting protection against a broad range of influenza virus strains. The long alpha helix (LAH) is an attractive vaccine component since it is one of the most conserved influenza hemagglutinin (HA) stalk regions. For an improved immune response, the LAH domain from H3N2 strain has been incorporated into virus-like particles (VLPs) derived from hepatitis B virus core protein (HBc) using recently developed tandem core technology.

**Results:**

Fermentation conditions for recombinant HBc-LAH were established in yeast *Pichia pastoris* and a rapid and efficient purification method for chimeric VLPs was developed to match the requirements for industrial scale-up. Purified VLPs induced strong antibody responses against both group 1 and group 2 HA proteins in mice.

**Conclusion:**

Our results indicate that the tandem core technology is a useful tool for incorporation of highly hydrophobic LAH domain into HBc VLPs. Chimeric VLPs can be successfully produced in bioreactor using yeast expression system. Immunologic data indicate that HBc VLPs carrying the LAH antigen represent a promising universal influenza vaccine component.

**Electronic supplementary material:**

The online version of this article (10.1186/s12896-017-0396-8) contains supplementary material, which is available to authorized users.

## Background

Worldwide, influenza epidemics result in excessive morbidity and the deaths of 250,000–500,000 people, annually [1]. Seasonal epidemics and pandemics are caused by group A influenza viruses, which are the main targets of seasonal vaccines. Currently licensed influenza vaccines are effective against homologous viruses, however, these vaccines need to be reformulated for every season on the basis of predictions of the upcoming circulating subtypes. Prediction mismatches can impact vaccine effectiveness, and have significant epidemiological and economical consequences [2]. Moreover, besides seasonal epidemics, influenza viruses can cause occasional pandemics. Thus, development of a universal influenza vaccine, which would protect against a broad spectrum of influenza viruses, is warranted. Novel attempts to construct such universal vaccines are generally based on conserved antigens from influenza matrix protein 2, as well as from the hemagglutinin (HA) stalk domain [3]. However, these antigens are naturally weak immunogens. Various strategies have been developed to elicit more potent immune responses with broader reactivity upon appropriate presentation to the host immune system [1, 4].

Presentation on the surface of symmetric virus-like particles (VLPs) stands out as one of the most efficient approaches to improve immune responses to weak antigens [5]. A number of attempts have been made to construct broadly protective candidate vaccines based on the conserved extracellular domain of matrix protein 2 (M2e) of influenza A viruses [6–9]. Although these M2e-based VLP vaccine candidates have been successfully tested in animal models, further efficacy studies are needed to demonstrate their protective potential in humans [1]. In addition, many scientists now believe that a universal and broad cross-protective influenza vaccine may require additional components. One such potential candidate is the structurally conserved 55 amino acid-long alpha helix (LAH) of the HA stalk domain.

Here, we incorporated the LAH domain from H3N2 influenza virus into HBc VLPs using novel tandem core technology based on the fusion of two HBc open reading frames to produce a single polypeptide chain. This strategy allows the insertion of large heterologous sequences in one of the two major immunodominant regions (MIR) located at the terminus of each spike, without compromising VLP formation [10]. A chimeric HBc-LAH gene was expressed in yeast *Pichia pastoris*. This expression system is preferred to bacteria because it avoids contamination with bacterial endotoxins. Fermentation and purification conditions compatible with industrial scale-up were established. The VLPs obtained induced a strong and broadly cross-reactive antibody response against several HA protein strains derived from both group 1 and group 2 influenza A virus subtypes in mice. These data indicate that tandem core VLPs carrying the LAH antigen represent a promising universal influenza vaccine component.

## Methods

### DNA constructions and yeast clone selection

A yeast codon optimized sequence encoding the tandem core construct including lysine linkers in both MIRs was designed and synthesized at GeneArt and cloned in the pPICZC vector (*Invitrogen*) using *Bst*BI and *Age*I restriction sites. Silent mutations were introduced up- and downstream of the two MIRs to create unique restriction sites (*Xba*I/*Not*I in Core 1 and *Eco*RI/*Nhe*I in Core 2). The resulting plasmid pPICZC PHe7 K1,K1 (K1-K1, for short) was used as a template for other MIR insertions. The lysine-encluding linker in Core 1 was replaced by yeast-optimized DNA sequence encoding the 55 amino acid long influenza H3N2 virus (A/Hong Kong/1/1968, Accession № AAK51718) HA stalk domain (corresponding to HA amino acids 420–474), using *Xba*I and *Not*I restriction sites. Resulting pPICZC coHe(LAH3,K1) construct (LAH3-HBc, for short) was linearized with *Pme*I to transform *P. pastoris* KM71H electrocompetent cells via electroporation. High copy number clones were selected in 96 well plates with liquid YPD medium containing 0.2 mg/mL (first 48 h) and 2 mg/mL (following 48 h) of zeocin. Cultures showing highest optical density (OD) at A = 600 nm were selected and spread on YPD agar plates in order to isolate single cell colonies. Selected clones were analysed for insert copy number by quantitative real time PCR based on the zeocin gene. The highest copy clone was selected and used to generate a Research Cell Bank.

### Fermentation conditions for LAH3-HBc

For seed culture, 2 × 250 mL of buffered glycerol-complex medium (BMGY; 1% (*w*/*v*) yeast extract, 2% (*w/v*) peptone, 100 mM potassium phosphate buffer pH 6.0, 1.34% (*w/v*) YNB, 0.0004% (*w/v*) biotin, and 1% (*v/v*) glycerol) was inoculated with 1.8 mL of cell bank suspension (BMGY culture in 30% (*v/v*) glycerol, OD = 25.0) in 2 L baffled Nalgene® shakeflasks. After 16–18 h the OD of the flask was between 15 and 20 and a 5% transfer volume was used to innoculate the bioreactor.

*Invitrogen’s* fermentation protocol for *Pichia pastoris* Mut^S^ strains was used to generate experimental material in a 30 L BIOSTAT Cplus bioreactor (Sartorius). The reactor was filled with Basal Salts medium (26.7 mL/L phosphoric acid (85%), 0.93 g/L CaSO_4_, 18.2 g/L K_2_SO_4_, 14.9 g/L MgSO_4_·7H_2_O, 4.13 g/L KOH, 40 g/L glycerol), plus 4.35 mL PTM_1_ trace salts per litre of Basal Salts media to achieve a total starting working volume of 10 L post-inoculation. The PTM1 trace salts contained: CuSO_4_·5H_2_O, 6.0 g/L; KI, 0.08 g/L; MnSO_4_·H_2_O, 3.0 g/L; Na_2_MoO_4_·2H_2_O, 0.2 g/L; H_3_BO_3_, 0.02 g/L; ZnCl_2_, 20.0 g/L; FeCl_3_, 13.7 g/L; CoCl_2_ · 6H_2_O, 0.9 g/L; H_2_SO_4_, 5.0 mL/L; and biotin, 0.2 g/L.

The bioreactor was run in batch-mode after inoculation. The dissolved oxygen tension (DOT) was maintained at 30% and was controlled in a sequence cascade by agitating the impeller between 400 to 1000 rpm followed by oxygen gas blending in ratio mode at a constant volumetric gas flowrate of 0.51 vvm. The pH range was maintained between 4.75–5.0 and pre-induction temperature at 30 ± 0.1 °C. A 20% drop in carbon evolution rate (CER) and spike in DOT, indicating depletion of carbon source, triggered the fed-batch induction phase. This was observed 28.5 h after bioreactor inoculation.

This fed-batch induction phase was maintained for 48 h at a fixed flowrate of 50 mL/h. The induction media itself is compromised of a 60:40 ratio of 50% (*v/v*) glycerol and pure methanol repectively, plus 12 mL PTM_1_ salts per liter of induction media. PPG2000 was used to prevent extensive foam formation throughout the fermentation. After 48 h of induction the culture was cooled to 12 °C to minimize proteolytic activity. Fermentation broth was harvested at 3000 *g*, 20 min and 4 °C. The wet pellets were weighed and stored at −20 °C.

### Purification and characterization of chimeric VLPs

Yeast cells were resuspended in lysis buffer (20 mM Tris HCl, 100 mM NaCl, 0.1% Triton X-100, pH = 8.0) at a proportion of 15% (*w/v*) and disrupted by French Press (4 cycles, 10,000 psi). The soluble fraction was separated by centrifugation (30 min, 18,000 *g*, +4 °C).

For purification of HBc K1-K1 VLPs, solid ammonium sulfate was added until 35% of saturation by continious stirring for 5 min following centrifugation (20 min, 18,000 *g*, +4 °C). The precipitate was dissolved in a minimal volume of 20 mM tris HCl pH = 8.0, subjected to thermal treatment (30 min at 55 °C) and centrifuged again. The supernatant was passed through an anion-exchange HiPrep 16/10 DEAE Fast Flow column and the flow-through fraction was collected. All chromatography runs were monitored and controlled by an ÄKTA FPLC chromatography device (GE Healthcare).

For purification of chimeric LAH3-HBc VLPs, PEG6000 50% solution in 20 mM Tris HCl, pH = 8.0 (*w/v*) was added dropwise to the cell supernatant under continious stirring until the final concentration reached 5% (*w/v*) and incubated 1 h at +4 °C. After centrifugation (20 min, 18,000 *g*, +4 °C) the precipitate was dissolved in a minimal volume of 20 mM tris HCl, 2 M urea and loaded onto a size-exclusion Sepharose 4 FF matrix (XK26/40 column, bed height 25 cm) in column buffer A (20 mM Tris HCl, 100 mM NaCl, 1 M urea, pH = 8.0) at V = 1 mL/min. 10 mL fractions were collected and analyzed by native agarose gel, denaturing PAGE, and electron microscopy. Selected fractions were pooled and loaded onto anion-exchange Fractogel EMD TMAE (M) column (Tricorn 10/50 column, 3.5 mL bed volume) equilibrated with column buffer A. Column-bound proteins were eluted with a linear gradient of 10 column volumes using column buffer A containing 1 M NaCl. Fractions containing VLPs were dialized (10 kDa MWCO membrane) against 100× excess of 20 mM Tris HCl, 100 mM NaCl, pH = 8.0, with two buffer exchanges, for 48 h at +4 °C. Dialized material was pooled and concentrated with Amicon 100 kDa MWCO filter until it reached concentration of about 1 mg/mL. The VLP preparation was aliquoted, slowly frozen at −70 °C in a Mr. Frosty™ container and used for immunization of mice.

Protein concentrations were estimated by the Bradford assay. The purity of protein samples was analyzed by SDS-PAGE according to standard protocols with a 4% stacking and 15% separating polyacrylamide gel (PAAG). To visualize protein bands, the gels were stained with Coomassie Brilliant Blue G-250. Alternatively, separated proteins were transferred onto nitrocellulose membrane and detected by immunoblotting with the monoclonal anti-HBc antibody and the anti-mouse IgG peroxidase conjugate (DAKO). To assess nucleic acid content, samples were subjected to native 1% agarose gel electrophoresis in TAE buffer (pH = 8.4) for approximately 0.5 h at 5 V/cm. Nucleic acids in the agarose gels were visualized by ethidium bromide staining. A 1 kb DNA ladder (Thermo Fischer Scientific) was used as a marker. For transmission electron microscopy, the protein samples (at c = 0.1–0.5 mg/mL) were adsorbed on carbon-Formvar-coated copper grids and negatively stained with 1% uranyl acetate aqueous solution. The grids were examined with a JEM-1230 electron microscope (JEOL Ltd., Tokyo, Japan) at 100 kV. Electron micrographs were recorded digitally using a side-mounted Morada camera (Olympus - Soft Imaging System GmbH, Munster, Germany) with iTEM software (version 3.2, Soft Imaging System GmbH).

### Mouse immunizations

All mouse experiments were performed in accordance with protocols approved by the Animal Welfare Structure of the Minister of Agriculture, Viticulture and the Consumer Protection of the Grand Duchy of Luxembourg (Ref. LNSI-2014-02). Female BALB/c mice were purchased from Harlan Laboratories, Inc. 8-week-old BALB/c mice were immunized using a standard protocol including 3 intraperitoneal injections at 2 weeks interval with 30 μg of chimeric LAH3-HBc VLPs for primary immunisation, and with twice 15 μg for the second and third injection. The antigen was dissolved in 100 μL phosphate buffered saline adjuvanted with 100 μL MF59 (Addavax, *Invivogen*) containing 20 μg CpG (OD2395 Vaccigrad, *Invivogen*) totaling 200 μL per injected dose.

### Enzyme-linked immunosorbent assay (ELISA)

Influenza-specific IgG in mouse serum was measured by indirect ELISA using the following purified recombinant group 1 and group 2 HA antigens at the given concentrations: A/Texas/36/1991 (Tex91) (H1, 1.5 μg/ml), A/California/4/2009 (Cal09) (H1, 3.5 μg/ml), A/Japan/305/57 (JP57) (H2, 1.25 μg/ml), A/Perth/16/2009 (Perth09) (H3, 0.625 μg/ml), A/Swine/Ontario/01911–1/99 (Onta99) (H4, 1.25 μg/ml), A/Vietnam/1203/04 (VN04) (H5, 1.25 μg/ml), A/Netherlands/219/2003 (Neth03) (H7, 2.5 μg/ml), A/Hong Kong/1073/99 (HK99) (H9, 1.25 μg/ml), A/Jiangxi-Donghu/346/2013 (JX13) (H10, 2.5 μg/ml), A/mallard /Alberta/294/1977 (Alb77) (H11, 1.25 μg/ml), A/mallard/Astrakhan/263/1982 (Astrak82) (H14, 1 μg/ml), and A/duck/AUS/341/1983 (AUS83) (H15, 2 μg/ml) all purchased from *Sino Biological Inc*. Subsaturating coating concentrations were determined with a serum of a mouse sublethally challenged either with pH1N1 (group 1 HA) or H3N2 (group 2 HA) or with specific monoclonal antibodies against HA H1N1, HA H3N2, HA H5N1, HA H7N9 and HA H9N2 from the same supplier, after coating ELISA plates with threefold dilution series of each antigen.

Wells of 384-well microtiter plates (Greiner) were coated overnight at 4 °C with 20 μL/well of 3.5 μg/mL purified HA proteins in carbonate buffer (100 mM, pH = 9.6) or with carbonate buffer alone as a background control. Irrelevant antigens (Cytomegalovirus grade 2 antigen, 2 μg/ml; purified EBV capsid protein, 30 ng/ml; Toxoplasma gondii antigen, 2 μg/ml; Microbix, Mississauga, Canada) served as negative antigen controls. Reactivity against “empty” HBc VLPs (20 μl per well of 2 μg/ml) was used as positive control. All subsequent steps were performed at room temperature. Wells were washed sequentially in washing buffer (Tris HCl containing 1% Tween 20) and blocked for 2 h with 1% BSA in Tris buffer. After washing, sera (starting with a 100-fold dilution) were added, incubated for 90 min, and washed. Alkaline phosphatase conjugated goat anti-mouse IgG (1/750 dilution, ImTec Diagnostics) was added for 90 min, washed and developed using 2-amino-2-methyl-1-propanol. Absorbance was measured at 405 nm (Spectromax Plus, Sopachem) after 60 min incubation.

## Results and discussion

### Modification of the HBc gene

The insertion of heterologous sequences into viral structural genes generates chimeric VLPs that can be used as stable nanocontainers for diagnostics, vaccination and gene transfer purposes [11, 12]. The successful assembly of chimeras is dependent on the nature of the insert as well as its position within the carrier gene. For HBc, the MIR is generally considered as the most promising insertion site of the HBc molecule due to its surface exposure and high insertion capacity [13]. Recently, a number of approaches have been developed for MIR-insertion and the presentation of “difficult” protein sequences including large, charged and/or hydrophobic domains, T-cell epitopes, and cell-receptor ligands. These strategies include the SplitCore [14], the use of a non-covalent “binding-tag” peptide [15], and the tandem core technology. The latter is based on a genetically fused HBc dimer that can assemble in correctly folded HBc VLPs [16] while accomodating large heterologous sequences [10].

**Fig. 1:**
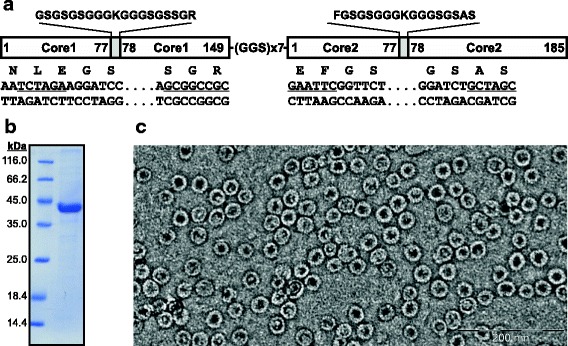
**a** Design of the HBc K1-K1 construct. Amino acid sequences of inserted lysine linkers are showed in upper part. Below, introduced unique restriction sites at both ends of the linkers (*Xba*I/*Not*I in Core1 and *Eco*RI/*Nhe*I in Core2) are underlined. Purified K1-K1 VLPs were characterized by Coomassie-stained PAAG (**b**) and electron microscopy (**c**)

Previously, we have demonstrated that the yeast *P. pastoris* system is well suited for high-level of synthesis and purification of wild-type HBc VLPs expressed from a cloned HBc monomer gene [17]. The current study is based on so called hetero-tandem core, where a C-terminally truncated HBc monomer gene (Core 1, 149 aa) was genetically fused via a GGSx7 linker with a full-length HBc monomer gene (Core 2, 185 aa) to form a covalently linked dimer. To further explore the potential of this HBc carrier, we introduced into each MIR two short sequences encoding a lysine codon flanked on both sides by flexible glycine linkers (Fig. 1a). Such surface-exposed lysine residues can be efficiently used for chemical coupling of peptides containing reactive SH groups provided by free cysteine residues [18]. The resulting construct K1-K1 was transformed in yeast *P. pastoris*. The selected clone containing multiple integrated expression units ensured high-level synthesis of the soluble target protein and was easily purified from yeast cells as correctly formed HBc VLPs (Fig. 1b-c).

**Fig. 2:**
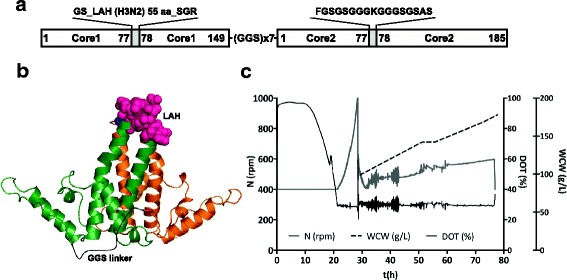
Construction and expression of the recombinant LAH3-HBc gene. Design (**a**) and cartoon (**b**) of the LAH3-HBc dimer. Individual HBc monomers are coloured in green and orange. First MIR contains the LAH domain (represented as random pink spheres) while the second MIR contains lysine linker (blue spheres). The model was created using PyMOL version 1.7rc1. The tandem core dimer was based on the structure from PDB-1QGT, with the linker added in black. **c** LAH3-HBc fermentation profile. Dissolved oxygen tension (DOT) was maintained above 30% through agitation control (N) between 400 to 1000 rpm. Biomass was monitored post-induction by wet cell weight (WCW) analysis

Our early attempts to incorporate a highly hydrophobic LAH domain from influenza HA stalk into the MIR of monomeric HBc gene in *E. coli* resulted in insoluble products (data not shown). This is in line with previous observations [19] and indicated that specific approaches like tandem core technology might be necessary to improve solubility and achieve formation of VLPs incorporating LAH. Since very promising results were obtained with the hetero-tandem HBc based K1-K1 construct, we inserted the LAH-encoding sequence into the MIR of the truncated HBc gene (Core 1) while the MIR of the full-length HBc (Core 2) remained unchanged (Fig. 2a-b). In order to exploit “endotoxin-free” cells with the potential for further industrial scale-up possibilities we transferred the chimeric tandem core gene into the yeast *P. pastoris* vector system resulting in LAH3-HBc construct.

### Expression of LAH3-HBc construct in yeast

Recently, a number of novel influenza monoclonal antibodies have been identified against the stalk domain of the HA relatively conserved across most HA subtypes. These antibodies are able to broadly neutralize a wide spectrum of influenza virus strains and subtypes [20]. The LAH domain is a small alpha-helical portion of the stalk domain which contains a well-characterised neutralizing epitope for mAb 12D1 [21]. However, when out of structural context LAH does not generate neutralizing antibodies. Nevertheless, several studies from different research groups suggest that the LAH domain is a promising vaccine component. The current hypothesis for the mechanism of action involves non-neutralising antibody mediated cellular immune processes such as antibody dependent cellular cytotoxicity and complement dependent cytotoxicity [22] which are difficult to measure in mice. The synthetic LAH peptide from H3N2 virus coupled to carrier protein keyhole limpet hemocyanin induced in mice not only strong protection against homologous virus but also partial protection against distinct virus strains [23]. Similarly, the LAH region of H7N9 influenza virus was inserted into the HBc gene and the resulting chimeric HBc-LAH VLPs induced strong protection against homologous and heterologous virus strains in mice [24]. The latter study, however, required a complex renaturation procedure extracting chimeric protein from insoluble aggregates in *E. coli*, a process that would be costly and difficult to scale-up for industrial manufacturing. In addition, this approach is also compromised by the presence of bacterial endotoxins.

To avoid these problems we aimed to produce LAH3-HBc VLPs in yeast cells. Yeast *P. pastoris* is well-adapted for large-scale fermentation and is a useful system for the expression of milligram-to-gram quantities of proteins for both basic laboratory research and industrial manufacture [25, 26]. Clones with multiply integrated expression cassettes were obtained in order to ensure maximal synthesis of the target protein in line with previous reports [27, 28]. Here, the clone with 50 ± 2 integrated expression units was selected for further experiments (see Additional file 1).

Fermentation conditions were established to maximise product yield in the soluble fraction, as determined by HBc-specific Western blots and its assembly into a VLPs, as determined by electron microscopy. It should be noted that several induction stategies were evalutated, including the standard methanol induction method for Mut^S^ strains. We observed that for the K1-K1 construct, the methanol only induction method yielded the most VLP material, yet when this same method was used for the LAH3-HBc construct, little or no product was observed (data not shown). The different constructs did not appear to effect biomass as both seemed to grow well. We therefore sought to examine different induction methods, including various flowrates and glycerol:methanol ratios in the induction feed. Eventually we discovered that the 60:40 ratio of 50% glycerol:methanol over 48 h of induction provided the best yield for LAH3-HBc. Based on the off-gas data (not shown), in all cases the cells seemed metabolically actively and grew well, however product expression was construct dependent – possibly indicating that the complexity and self-assembly of different VLP constructs are not intrinsically linked to cell metabolism during fermentation. Wet cell weight at the end of fermentation reached 178 g/L. The overall fermentation process is presented schematically in Fig. 2c.

### Purification and characterization of chimeric LAH3-HBc VLPs

**Fig. 3:**
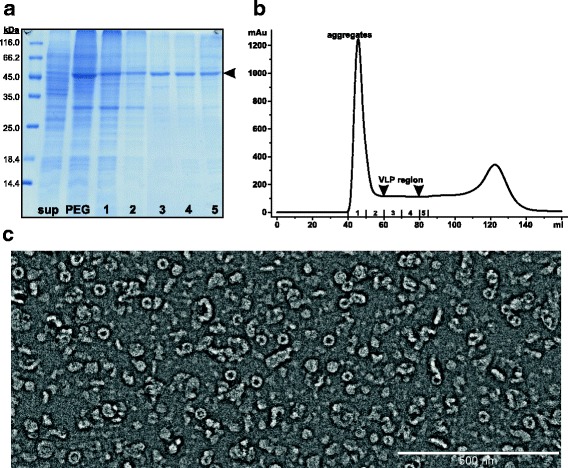
Extraction of the LAH3-HBc protein from yeast cell lysates. **a** Coomassie-stained PAAG illustrating individual purification steps. Target protein corresponding band is marked by arrowhead. Sup, protein content in cell supernatant; PEG, protein precipitate loaded onto SEC column; 1–5, SEC fractions corresponding to column profile (**b**). **c** Electron microscopy analysis of VLP region from SEC elution

Efficient and selective concentration of the protein of interest from crude cell lysates is often a critical step determining overall success of the whole purification process. For HBc VLPs, ammonium sulfate precipitation has been used with good results ([17] and references herein). However, in this particular case it resulted in co-precipitation of the VLPs with other host cell proteins which were difficult to remove in the subsequent purification steps (data not shown). In contrast, addition of PEG led to selective enrichment of the target protein with a calculated MW of 48.2 kDa from cell supernatant (Fig. 3a). Since the LAH domain is highly hydrophobic, addition of urea was necessary to complete solubilization of the PEG precipitate and reduce aggregation caused by hydrophobic interactions. Next, the protein mixture was loaded onto a size-exclusion chromatography (SEC) column containing Sepharose 4 FF, a matrix developed for industrial processing of large molecules and virus particles at high flow rates and moderate pressures. We did not exceed the bed height of 25 cm in line with manufacturer’s recommendations. A typical chromatography profile is shown in Fig. 3b. The aggregate peak appears first and this also contains a minor proportion of the target protein. The peak fractions are cloudy, contain a large amount of contaminating proteins and are not useful for the further purification of LAH3-HBc VLPs (data not shown). The next part of the profile is the so called “flat” region which theoretically corresponds to migration of VLP-size proteins. Indeed, SDS-PAGE analysis revealed the presence of LAH3-HBc sized protein in these fractions (Fig. 3a) while electron microscopy confirmed the presence of correctly formed VLPs (Fig. 3c). However, the major component of these fractions comprised misassembled but soluble protein material.

**Fig. 4:**
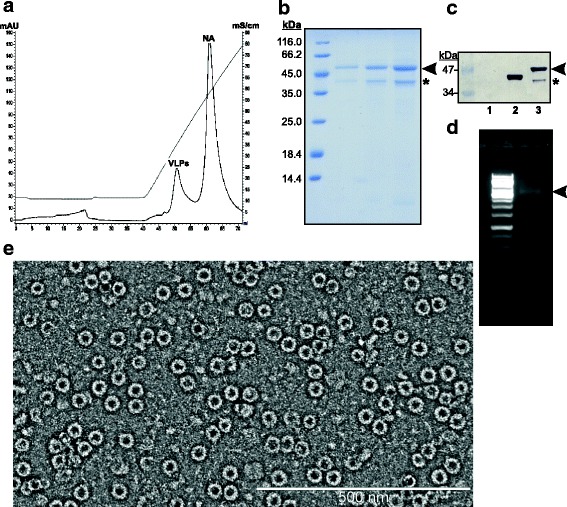
Final purification (**a**) and characterization (**b**-**e**) of LAH3-HBc VLPs. **a** Fractogel TMAE chromatography profile. VLP and free nucleic acid (NA) peaks are indicated. **b** Coomassie-stained PAAG of final product after dialysis and concentration. 1, 2 and 4 μl of protein are loaded per track. **c** Western blotting of final product. Lane 1, negative control; lane 2, K1-K1 VLPs; lane 3, LAH3-HBc VLPs. Full-length protein and product related bands are marked by arrowhead and asterix, respectively. **d** Nucleic acid content of final product in native agarose gel. Visible is only VLP-associated NA band (indicated by arrowhead). **e** Quality of LAH3-HBc VLPs verified by electron microscopy

For the second stage of the purification we aimed to maximally reduce the content of (i) contaminating proteins, (ii) misassembled LAH3-HBc aggregates and (iii) VLP non-associated nucleic acid. Ideally, this should be achieved by a single chromatography step. Our attempts to use either Sephacryl S1000 SEC, or differential precipitation of fractions by ammonium sulfate/PEG generally failed due to low yield and/or poor reproducibility (data not shown). We then subjected the protein solution to several anion exchangers as a relatively cheap, robust material capable of withstanding harsh cleaning-in-place conditions. The strong anion exchanger Sepharose Q HP matrix ensured good separation of nucleic acid but led to disassembly of VLPs (data not shown). Experiments were continued with Fractogel DEAE and TMAE matrices representing weak and strong anion exchangers, respectively. Both of them resulted in significant enrichment of correctly folded VLPs in column-eluted material, however, only the TMAE matrix ensured nearly complete separation of VLPs from VLP-free nucleic acid (Fig. 4a). After dialysis and concentration the final product was analyzed for its purity, nucleic acid content and VLP quality (Fig. 4b-e). The final product reached at least 90% of homogeneity but appeared as two bands in PAAG (Fig. 4b). However, both bands reacted in Western blotting with anti-HBc antibody (Fig. 4c) indicating that the lower one is not a contaminant but a product related band. Since this protein was not removed by SEC we assume it is incorporated into the VLPs.

After the first SEC column, the total protein content of VLP region was calculated as ~4 mg per 5 g of wet cells. These fractions, however, contained a mixture of proteins with an indeterminate percentage of correctly folded VLPs. In contrast, after elution from the anion exchange column more than 80% of material was in the form of VLPs but the yield in terms of total protein was decreased to ~0.5 mg. This result is in line with the assumption that correctly assembled VLPs comprise about 10% of total protein after SEC separation. These data were reproduced with consistency in at least three independent purification processes.

To summarize, in conditions described a final yield of LAH3-HBc VLPs could be estimated as 17–18 mg/L. For comparison, outcome of K1-K1 VLPs is typically ~0.8 mg per 1 g of cells, with its biomass after fermentation 260–270 g/L, which gives final yield 208–216 mg/L. Therefore incorporation of LAH domain decreases outcome of VLPs by more than the order of magnitude. This is still acceptable for industrial manufacturing, although further optimisation may increase the outcome and lower the process cost.

### Induction of broadly reactive antibodies by immunization

**Fig. 5:**
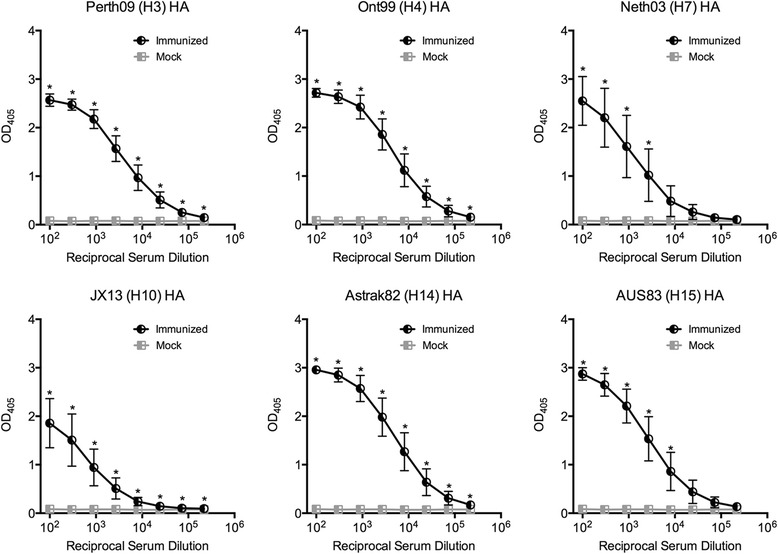
Reactivity of mouse sera with recombinant group 2 HA proteins (H3, H4, H7, H10, H14 and H15). Mice were immunized with LAH3-HBc VLPs or adjuvant alone (“Mock”). The panels show the mean and standard deviation of the optical density at 405 nm of the sera of 16 mice per group and are representative of two distinct experiments

**Fig. 6:**
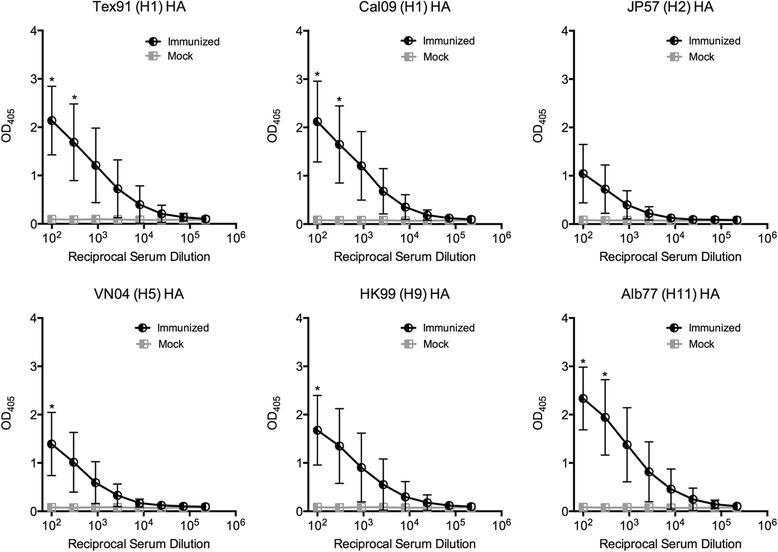
Reactivity of mouse sera with recombinant group 1 HA proteins (seasonal HA1, pandemic H1, H2, H5, H9 and H11). The mouse sera were the same as for Fig. 5. The panels show the mean and standard deviation of the optical density at 405 nm of the sera of 16 mice per group and are representative of two distinct experiments

**Fig. 7:**
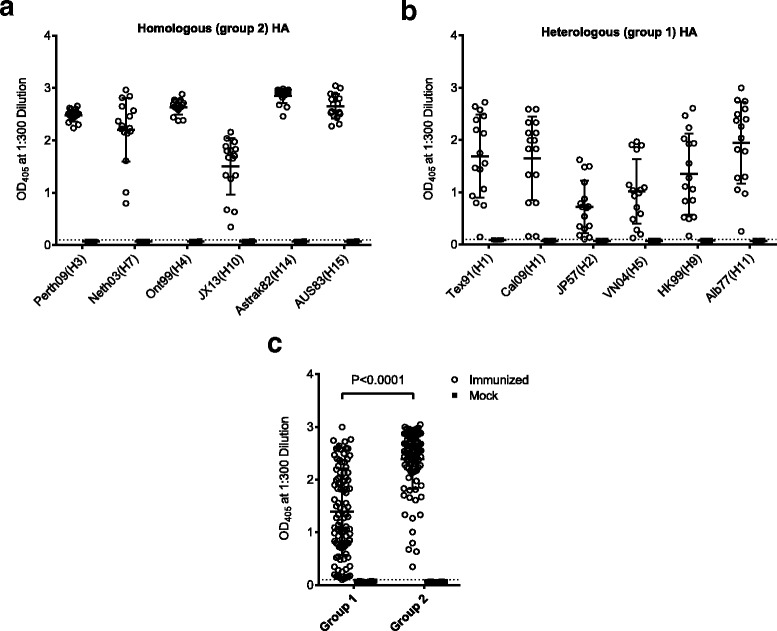
Reactivity of individual mouse sera (OD405 of a 1:300 serum dilution from Figs. 5 and 6) with homologous group 2 (**a**) and heterologous group 1 HA antigens (**b**) after immunization with LAH3-HBc VLPs (*N* = 16) or after mock (*N* = 16) immunization. **c** shows the same aggregated results by reactivity with group 1 or group 2 antigens. The threshold for positivity (dotted line) is defined by comparison to the mock i.e. adjuvant only immunized mice

To elicit antibodies against the influenza virus, BALB/c mice were immunized 3 times with chimeric LAH3-HBc VLPs at two weeks intervals. 10 days after the third immunization all immunized animals showed high titers of antibodies against the homologous group 2 HA proteins, including H3, H4, H7, H10, H14, and H15, while the mock group showed no reactivity with the antisera. Only H10 gave a weaker reaction (Fig. 5). In addition, the VLP-induced antisera cross-reacted significantly with the heterologous group 1 HA proteins, including H1, H2, H5, H9, and H11, but the reactivity was significantly weaker (*P* < 0,0001) than with group 2 HA (Figs. 6 and 7). The lowest reactivity was with H2 and H5 antigens. Fig. 7 shows that all mice reacted at a serum dilution of 1:300 with all group 2 HA species. Those with the lowest reactivity to the group 2 HA antigen were also the same one or two mice which had consistantly low levels of reactivity with group 1 HA. Thus chimeric LAH3-HBc VLPs induced broadly reactive anti-influenza serum against both group 1 and 2 HA proteins. While all animals reacted with the “empty” K1-K1 HBc VLPs, none of them showed any reactivity (OD < 0,1) with the irrelevant antigens (EBV, CMV, Toxoplasma, see Additional file 2).

## Conclusion

We have demonstrated that the tandem core technology is a useful tool for incorporation of “difficult” sequences like the hydrophobic LAH domain into HBc VLPs while cloning into the classical, unmodified monomeric HBc gene results in insoluble products. The chimeric LAH3-HBc VLPs were successfully produced in a bioreactor using yeast *P. pastoris* expression system. The relatively low production yield was partially compensated by an efficient purification protocol of the chimeric VLPs. The LAH3-HBc VLPs induced broadly reactive antibodies against both group 1 and 2 HA proteins, the hallmark of a universal vaccine against influenza A viruses.

## Additional files

Additional file 1: Determination of copy number for integrated expression units in selected *P. pastoris* clones. Description of the methodology used for quantification of copy number for expression cassettes integrated in selected *P. pastoris* clones. The method is based on real time PCR amplification of zeocin gene. (DOCX 186 kb)

Additional file 2: Serum IgG reactivity against the “empty” K1-K1 HBc VLPs and against irrelevant antigens (Cytomegalovirus antigen, purified EBV capsid protein, Toxoplasma gondii antigen). Reactivity of mouse sera with carrier HBc-derived VLPs (positive control) in comparison with irrelevant antigens (negative control) demonstrating specificity of ELISA method used. (PPTX 91 kb)

## Abbreviations

HA: Hemagglutinin
HBc: Hepatitis B virus core protein
LAH: Long alpha helix
MIR: Major immunodominant region
OD: Optical density
PAAG: Polyacrylamide gel
SEC: size exclusion chromatography
VLPs: Virus-like particles
