# Supplementary material for: Production and purification of chimeric HBc virus-like particles carrying influenza virus LAH domain as vaccine candidates
Source: BMC Biotechnol. 2017 Nov 10;17:79. doi: 10.1186/s12896-017-0396-8 (PMC5681787; doi:10.1186/s12896-017-0396-8)
Supplement: Supplementary file 1 — Determination of copy number for integrated expression units in selected P. pastoris clones. Description of the methodology used for quantification of copy number for expression cassettes integrated in selected P. pastoris clones. The method is based on real time PCR amplification of zeocin gene. (DOCX 186 kb) [file 12896_2017_396_MOESM1_ESM.docx]

Determination of copy number for integrated expression units

in selected *P. pastoris* clones

In order to establish the number of inserted copies integrated into the yeast genome, qPCR assay was performed on 8 single cell-colony *P. pastoris* KM71H/LAH3-HBc clones targeting the zeocin gene integrated along with the each expression cassette. Amplification primers for qPCR were as follows:

Zeocin-F: 5'- AGGTGAGGAACTAAACCATGGC -3'

Zeocin-R: 5'- TTGTCCGGCACCACCTGGT -3'

***Sample preparation***

- For each clone, genomic DNA was extracted and concentration was adjusted to 10 ng/µL with dH_2_O
- Zeocin gene was used as a standard. It was PCR-amplified using the following primers:

Zeocin std-F: 5'- AAGCATAGCAATCTAATCTAAGG -3'

Zeocin std-R: 5'- TTCGTGGACACGACCTCCGACC -3'

- Eight DNA concentrations of amplified zeocin gene from 10^-1^ to 10^-8^ ng/µL were prepared. In particular, the DNA concentration (of the PCR product) was checked using Spectra MAX 190 plate reader (Molecular Devices) and adjusted to 0.1 ng/µL (2.65*10^8^ copies per µL). This was then used as highest concentration to make serial 1:10 dilutions (from 1*10^-1^ to 1*10^-8^ ng/µL corresponding to 2.65*10^8^ and 2.65*10^1^ copies of zeocin PCR product, respectively)

***Reaction conditions***

- The qRT-PCR analysis was based on the detection and amplification of zeocin gene incorporated into the yeast genome using 2X QuantiFast SYBR Green PCR Kit (Qiagen)
- For each reaction 12.5 µL of master mix (HotStarTaq Plus DNA Polymerase, QuantiFast SYBR Green PCR Buffer, SYBR Green I dye, ROX dye and dNTP mix), 0.25 µL of 10 µM forward primer stock (Zeocin-F, final concentration 0.1 µM), 0.25 µL of 10 µM reverse primer stock (Zeocin-R, final concentration 0.1 µM), 1 µL of DNA template and 11 µL of water for a final volume of 25 µL
- As negative control water instead of template was used
- All reactions were performed in triplicates in Hard-Shell Low-Profile Thin-Wall 96-Well Skirted PCR Plates (BIO-RAD)
- Thermocycling conditions were as follows: 1) 95 °C for 10 minutes, 2) 95 °C for 10 seconds, 3) 55 °C for 30 seconds. Steps 2 and 3 were repeated 39 times. Reactions were performed using a CFX Connect Real-Time System PCR thermocycler (BIO-RAD)

Results are shown in Figure 1.


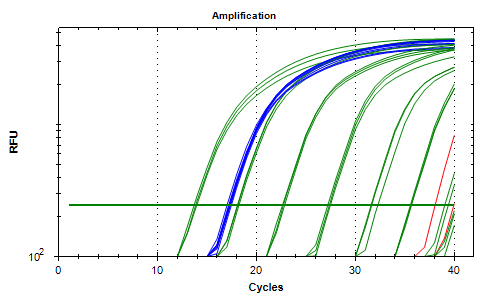


**Figure 1.** qRT-PCR amplification plot showing the amplification curves for the standards and the clones analysed. Green curves represent the standards, blue curves are the LAH3-HBc samples and red curves are the negative control. Samples were ran in triplicates.

***Data analysis***

- The starting quantities used for the standard samples ranged from 2.65*10^8^ to 2.65*10^1^ copies of zeocin PCR product. A standard curve was built based on the Ct values of each zeocin standard sample correlating with their starting concentrations (gene copy number) using BioRad CFX software (BIO-RAD; Fig. 2)
- The Ct values of each sample were compared with the standard curve and the insert copy number was extrapolated using the equation obtained from the standard curve. In this way, the number of copies present in each sample was established
- The copy number was then divided by the copies of yeast genomes present in the amount of template used (9.65*10^5^ per 10 ng) to give the zeocin copy number in each genome for each clone and consequently the copy number of insert


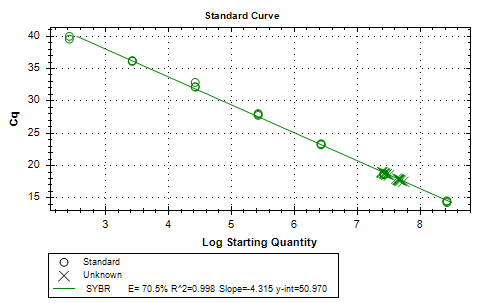


**Figure 2.** Generation of standard curve from qRT-PCR amplification of the samples. Circles represent zeocin controls and crosses represent the LAH3-HBc samples. The equation obtained was used for determination of the copy number in each sample.

In such way the clone with maximum number of integrated expression units has been selected and used for creation of Research Cell Bank. Results indicate 47, 52 and 48 copies of LAH3-HBc insert integrated into the yeast genome when analyzed at different stages of cultivation (Fig. 3). These data show high reproducibility and indicate that the insert copy number of LAH3-HBc for selected clone is approximately 50.

**Figure 3.** Estimation of LAH3-HBc copy number integrated in P. pastoris genome for the selected highest copy number clone. Results obtained are 47 (stock before freezing), 52 (stock after one day at -80 °C) and 48 (stock after six days at -80 °C).

*Proof of sequence*

In order to verify that correct gene has been integrated into *P. pastoris* genome, a full LAH3-HBc encoding sequence was amplified from genomic DNA of selected highest copy clone using primers:

Pic_upstream_F (5'- TGAACCCCGGTGCACCTGTGC -3') and

Pic_downstream_Rv (5'- TCTGAAGAGGAGTGGGAAATACC -3').

PCR products were then analysed on 1% (w/v) agarose gel (Fig. 4), purified and sent for sequencing to GATC Biotech (<https://www.gatc-biotech.com/en/index.html>) following the company instructions for sample preparation. Sequence alignments showed the exact match between expected and cloned nucleotide sequences. Proof of sequence was thus established.


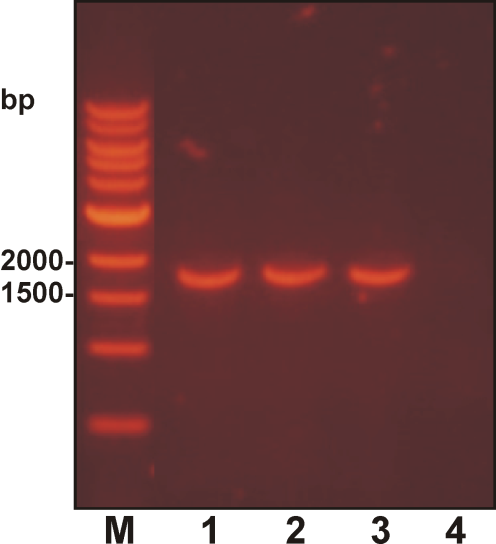


**Figure 4.** PCR products obtained after LAH3-HBc amplification. DNA was amplified from selected highest-copy clone at different stages of cultivation. The bands obtained appear to be of the expected molecular weight of 1950 bp. M: 1 kb DNA ladder; lane 1, LAH3-HBc single colony stock PCR product; lane 2, LAH3-HBc stock before storage; lane 3, LAH3-HBc stock after storage at -80 °C; lane 4: LAH3-HBc negative control.
