# Supplementary material for: Production and purification of chimeric HBc virus-like particles carrying influenza virus LAH domain as vaccine candidates
Source: BMC Biotechnol. 2017 Nov 10;17:79. doi: 10.1186/s12896-017-0396-8 (PMC5681787; doi:10.1186/s12896-017-0396-8)
Supplement: Supplementary file 2 — Serum IgG reactivity against the “empty” K1-K1 HBc VLPs and against irrelevant antigens (Cytomegalovirus antigen, purified EBV capsid protein, Toxoplasma gondii antigen). Reactivity of mouse sera with carrier HBc-derived VLPs (positive control) in comparison with irrelevant antigens (negative control) demonstrating specificity of ELISA method used. (PPTX 91 kb) [file 12896_2017_396_MOESM2_ESM.pptx]

## Slide 1
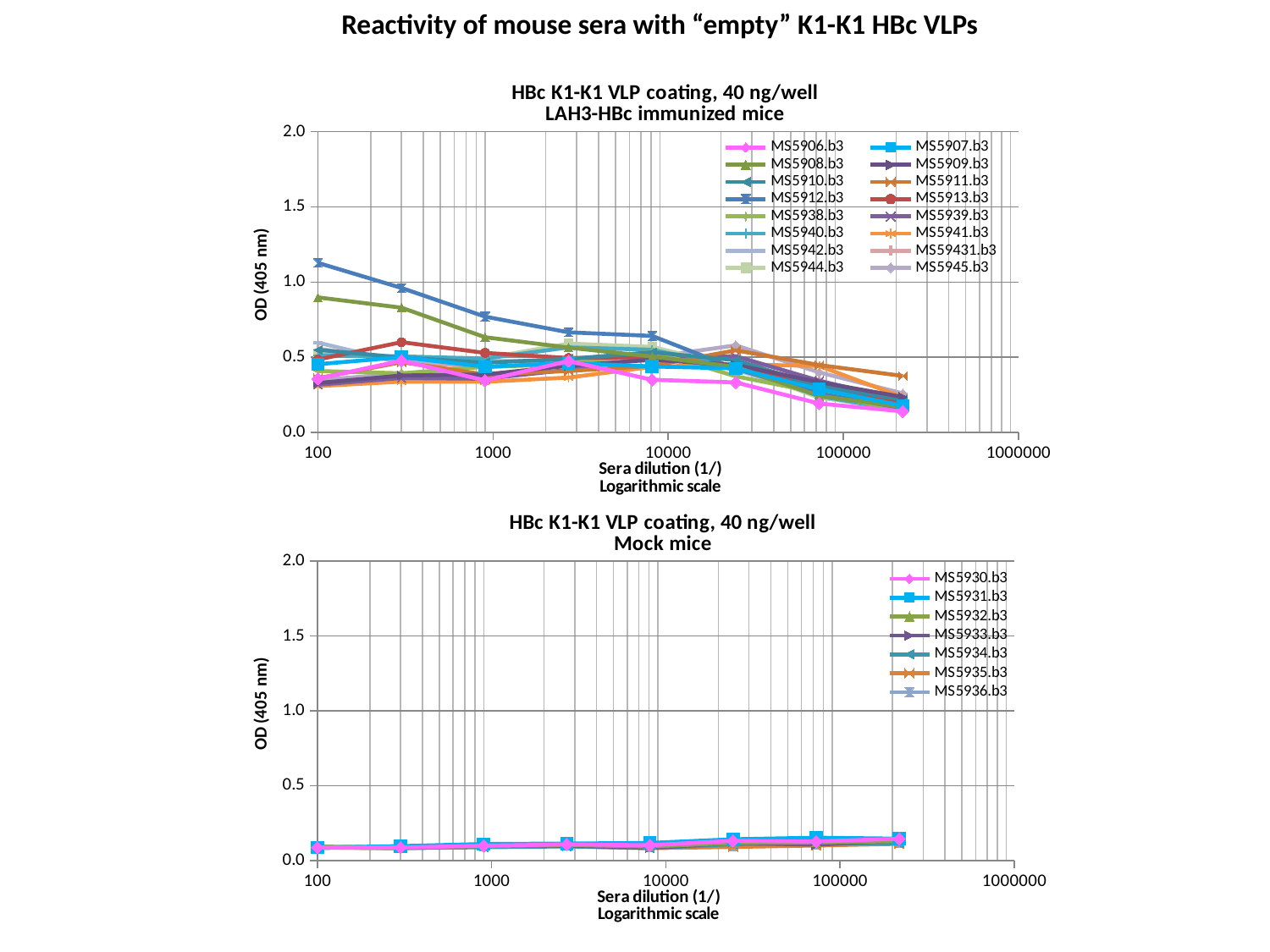

Reactivity of mouse sera with “empty” K1-K1 HBc VLPs
### Chart: HBc K1-K1 VLP coating, 40 ng/well
LAH3-HBc immunized mice
| Category | MS5906.b3 | MS5907.b3 | MS5908.b3 | MS5909.b3 | MS5910.b3 | MS5911.b3 | MS5912.b3 | MS5913.b3 | MS5938.b3 | MS5939.b3 | MS5940.b3 | MS5941.b3 | MS5942.b3 | MS59431.b3 | MS5944.b3 | MS5945.b3 |
|---|---|---|---|---|---|---|---|---|---|---|---|---|---|---|---|---|
### Chart: HBc K1-K1 VLP coating, 40 ng/well
Mock mice
| Category | MS5930.b3 | MS5931.b3 | MS5932.b3 | MS5933.b3 | MS5934.b3 | MS5935.b3 | MS5936.b3 |
|---|---|---|---|---|---|---|---|

## Slide 2
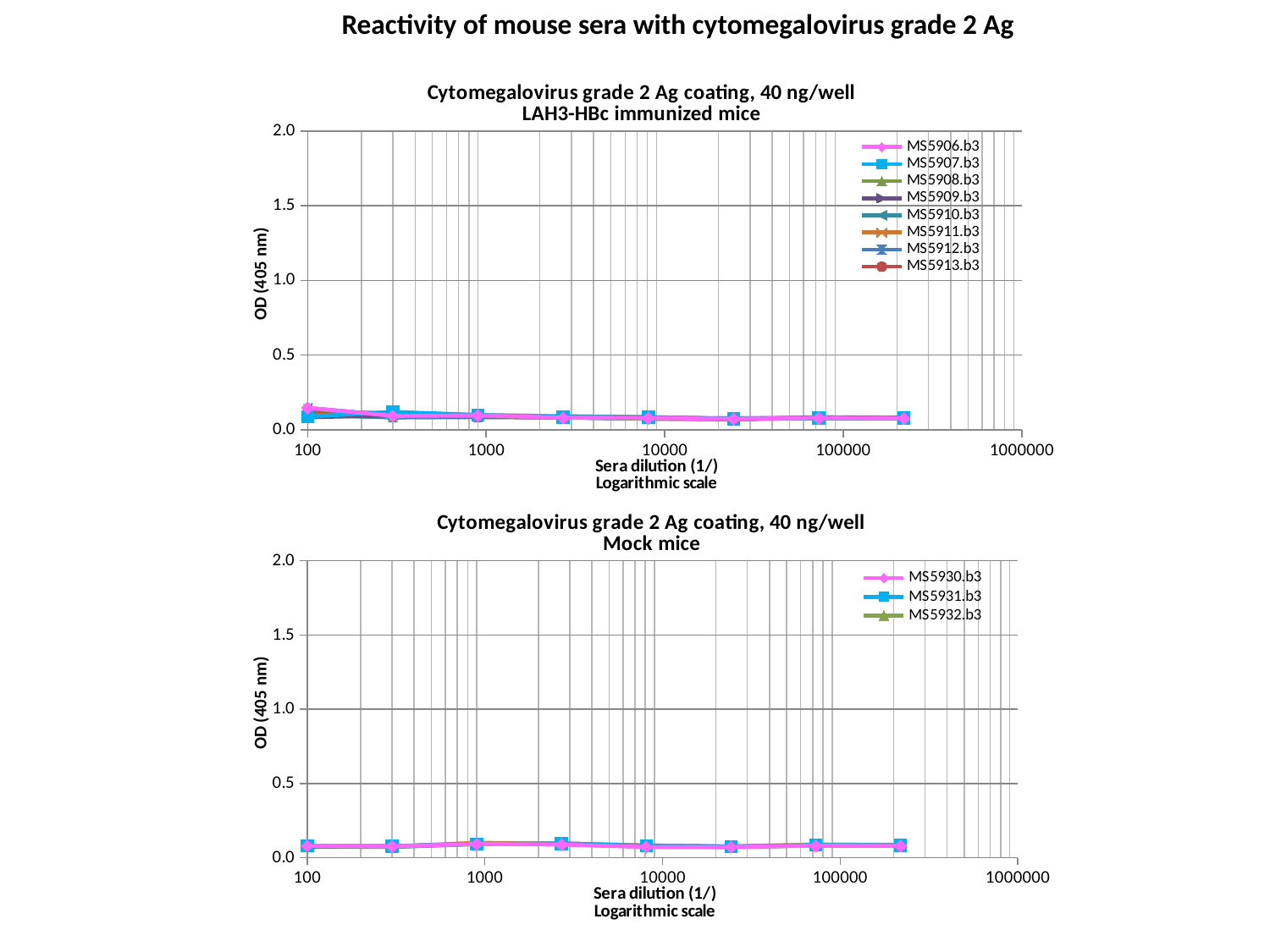

Reactivity of mouse sera with cytomegalovirus grade 2 Ag
### Chart: Cytomegalovirus grade 2 Ag coating, 40 ng/well
LAH3-HBc immunized mice
| Category | MS5906.b3 | MS5907.b3 | MS5908.b3 | MS5909.b3 | MS5910.b3 | MS5911.b3 | MS5912.b3 | MS5913.b3 | MS5938.b3 | MS5939.b3 | MS5940.b3 | MS5941.b3 | MS5942.b3 | MS59431.b3 | MS5944.b3 | MS5945.b3 |
|---|---|---|---|---|---|---|---|---|---|---|---|---|---|---|---|---|
### Chart: Cytomegalovirus grade 2 Ag coating, 40 ng/well
Mock mice
| Category | MS5930.b3 | MS5931.b3 | MS5932.b3 | MS5933.b3 | MS5934.b3 | MS5935.b3 | MS5936.b3 |
|---|---|---|---|---|---|---|---|

## Slide 3
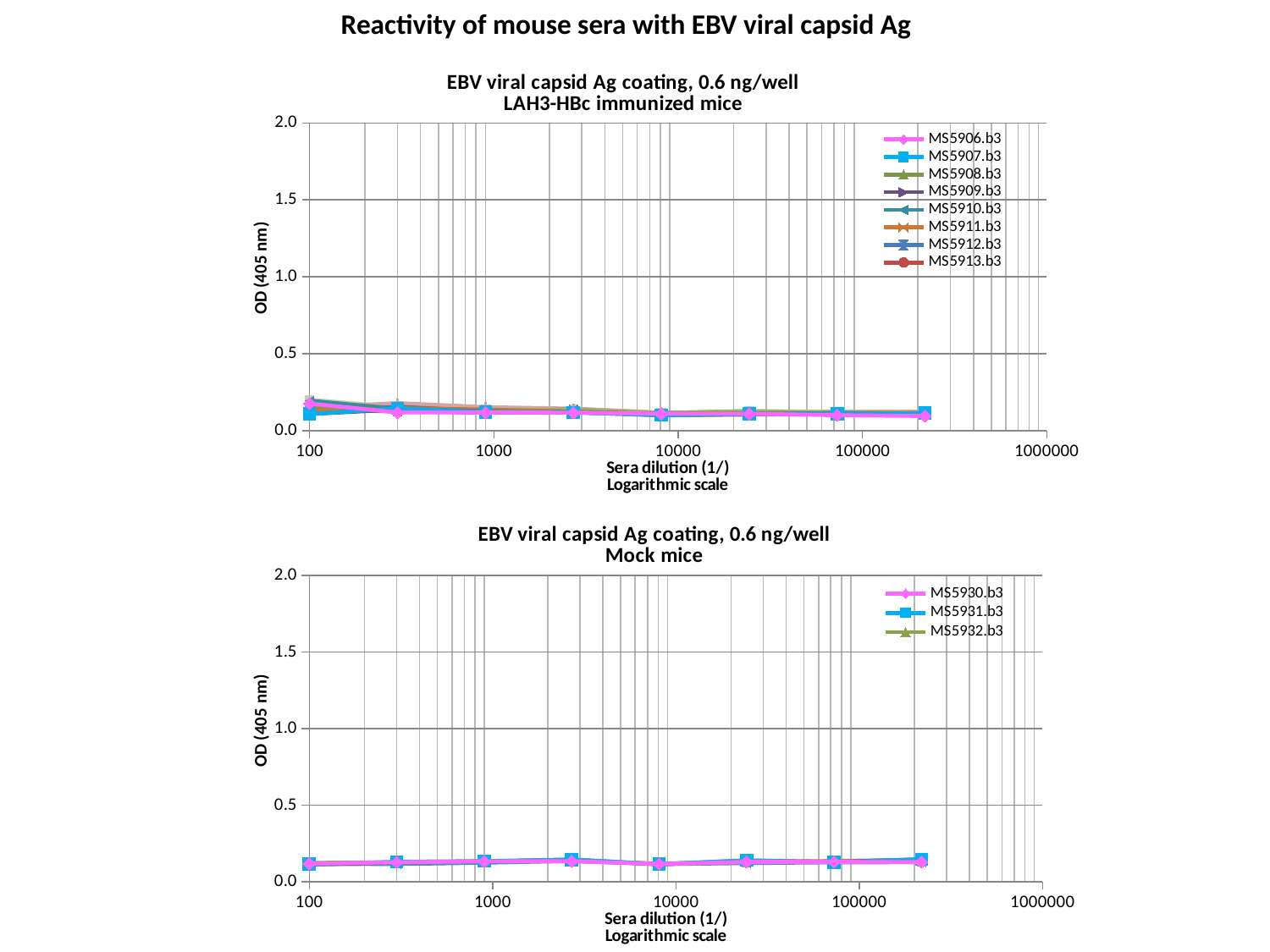

Reactivity of mouse sera with EBV viral capsid Ag
### Chart: EBV viral capsid Ag coating, 0.6 ng/well
LAH3-HBc immunized mice
| Category | MS5906.b3 | MS5907.b3 | MS5908.b3 | MS5909.b3 | MS5910.b3 | MS5911.b3 | MS5912.b3 | MS5913.b3 | MS5938.b3 | MS5939.b3 | MS5940.b3 | MS5941.b3 | MS5942.b3 | MS59431.b3 | MS5944.b3 | MS5945.b3 |
|---|---|---|---|---|---|---|---|---|---|---|---|---|---|---|---|---|
### Chart: EBV viral capsid Ag coating, 0.6 ng/well
Mock mice
| Category | MS5930.b3 | MS5931.b3 | MS5932.b3 | MS5933.b3 | MS5934.b3 | MS5935.b3 | MS5936.b3 |
|---|---|---|---|---|---|---|---|

## Slide 4
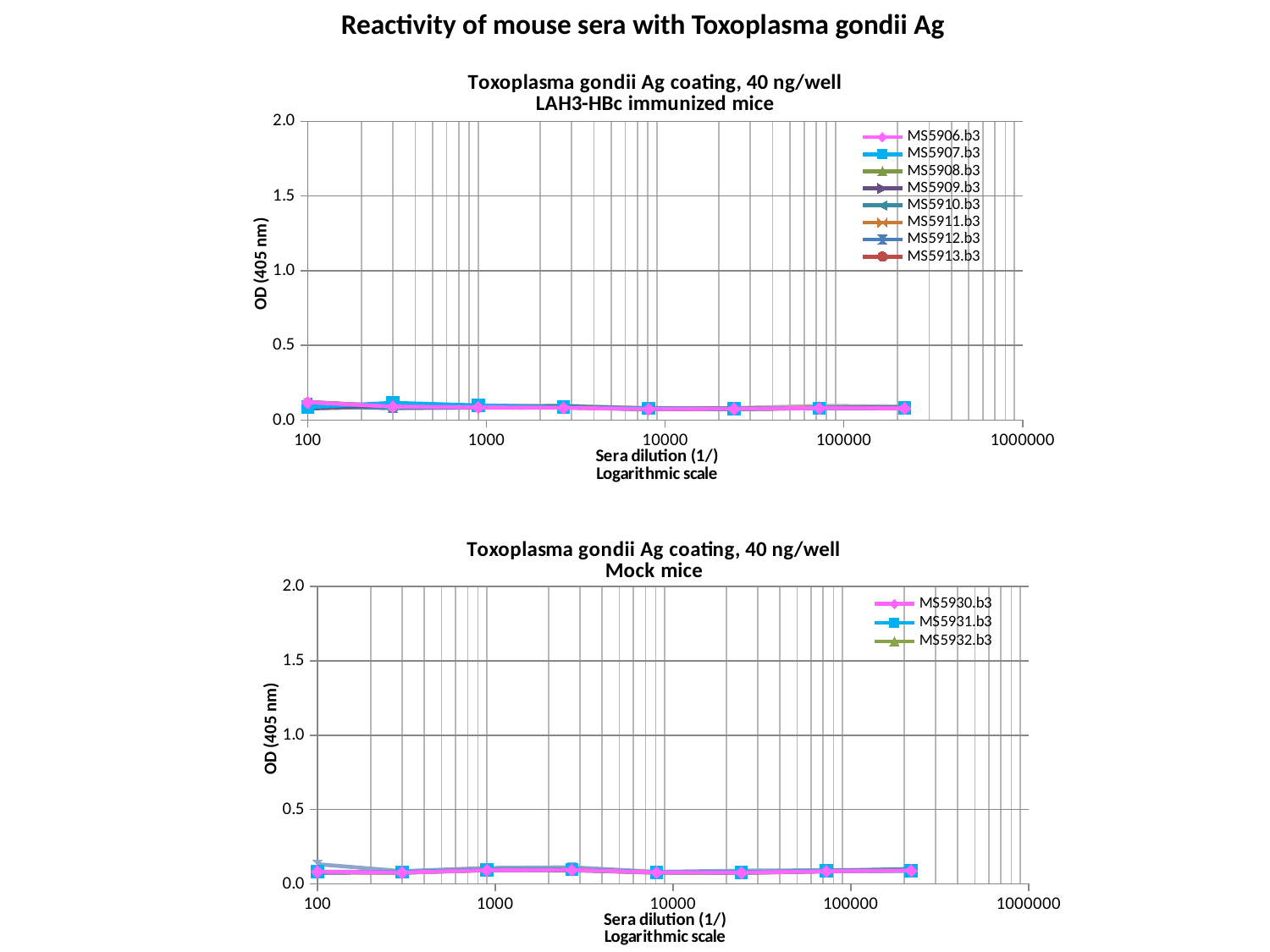

Reactivity of mouse sera with Toxoplasma gondii Ag
### Chart: Toxoplasma gondii Ag coating, 40 ng/well
LAH3-HBc immunized mice
| Category | MS5906.b3 | MS5907.b3 | MS5908.b3 | MS5909.b3 | MS5910.b3 | MS5911.b3 | MS5912.b3 | MS5913.b3 | MS5938.b3 | MS5939.b3 | MS5940.b3 | MS5941.b3 | MS5942.b3 | MS59431.b3 | MS5944.b3 | MS5945.b3 |
|---|---|---|---|---|---|---|---|---|---|---|---|---|---|---|---|---|
### Chart: Toxoplasma gondii Ag coating, 40 ng/well
Mock mice
| Category | MS5930.b3 | MS5931.b3 | MS5932.b3 | MS5933.b3 | MS5934.b3 | MS5935.b3 | MS5936.b3 |
|---|---|---|---|---|---|---|---|
